# The Combined Effects of Aspartame and Acesulfame-K Blends on Appetite: A Systematic Review and Meta-Analysis of Randomized Clinical Trials

**DOI:** 10.1093/advances/nmac072

**Published:** 2022-09-03

**Authors:** Kirnjot Mehat, Yi Chen, Christopher Peter Corpe

**Affiliations:** Department of Nutritional Sciences, Faculty of Life Sciences and Medicine, School of Life Courses, King's College London, London, United Kingdom; Department of Nutritional Sciences, Faculty of Life Sciences and Medicine, School of Life Courses, King's College London, London, United Kingdom; Department of Nutritional Sciences, Faculty of Life Sciences and Medicine, School of Life Courses, King's College London, London, United Kingdom

**Keywords:** nonnutritive sweeteners, aspartame, acesulfame-K, human interventional studies, energy intake, appetite, blood glucose, incretins

## Abstract

Aspartame (Asp) and acesulfame-K (Ace-K) are nonnutritive sweeteners (NNSs) commonly used in combination to replace added sugars in reduced- or low-calorie foods and beverages. Despite Asp/Ace-K blends having negligible calories, their effects on appetite have not been reviewed systematically. We therefore undertook a systematic review and meta-analysis of the metabolic effects of Asp/Ace-K blends on energy intake (EI), subjective appetite scores, blood glucose, and the incretin hormones glucose-dependent insulinotropic peptide and glucagon-like peptide. MEDLINE, Web of Science, and Cochrane CENTRAL databases (Embase, PubMed, and CINAHL) were searched (May 2021) for randomized controlled trials (RCTs). Human RCTs using Asp/Ace-K blends compared with sugar and water controls were included, whereas isolated cell and animal studies were excluded. An overall 4829 publications were identified and 8 studies, including 274 participants, were retrieved for review. The Asp/Ace-K group's EI was significantly reduced compared with sugar [mean difference (MD): –196.56 kcal/meal; 95% CI: –332.01, –61.11 kcal/meal; *P* = 0.004] and water (MD: –213.42 kcal/meal; 95% CI: –345.4, –81.44 kcal/meal; *P* = 0.002). Meta-analysis of subjective appetite scores and incretins could not be undertaken due to inconsistencies in data reporting and insufficient data, respectively, but of the 4 studies identified, no differences were observed between Asp/Ace-K blends and controls. The Asp/Ace-K group's blood glucose was nonsignificantly reduced compared with sugar (MD: –1.48 mmol/L; 95% CI: –3.26, 0.3 mmol/L; *P* = 0.1) and water (MD: –0.08 mmol/L; 95% CI: –0.62, 0.47 mmol/L; *P* = 0.78). Lower EI in participants who were predominantly healthy and assigned to Asp/Ace-K blends could not be reliably attributed to changes in subjective appetite scores. Blood glucose and incretins were also generally not affected by Asp/Ace-K blends when compared with controls. Additional short- and long-term RCTs using NNSs and sugars at dietarily relevant levels are needed. This trial was registered at the International Prospective Register of Systematic Reviews (PROSPERO: CRD42017061015).

## Teaser

**Statement of Significance**: In this report, we have undertaken a systematic review and meta-analysis of human appetite studies that used combinations of the NNSs aspartame and acesulfame-K, which are commonly used in foods and beverages. We found that EI is lower in Asp/Ace-K blend consumers when compared with controls; however, we could not reliably attribute changes in EI to changes in subjective appetite scores, gut peptides, or blood glucose. Higher-quality short- and long-term studies are therefore warranted.

## Introduction

Nonnutritive sweeteners (NNSs) such as aspartame (Asp), acesulfame-K (Ace-K), sucralose, saccharin, and stevia provide a replacement for sucrose in foods and beverages and, due to their negligible caloric content, have been suggested to be a way of reducing energy intake (EI) without compromising sweet taste (1). However, some researchers have proposed that NNSs may interfere with learned responses to sugar-like foods via activation of the tongue's sweet taste receptor (T1R2/T1R3) resulting in central nervous system signaling (cephalic responses) or activation of the reward systems, which may spur cravings for energy-dense foods, increasing appetite and food intake (2, 3). The effects of NNSs in mammals have been extensively reviewed (4, 5), and findings have given rise to conflicting views on their metabolic effects. For example, mice fed saccharin over a period of weeks had significantly increased EI and weight gain when compared with glucose-fed controls (6, 7). However, these findings were not replicated in rats (8), and evidence from short-term (≤1 d) and sustained (weeks to months) human interventional studies showed the opposite effect: when compared with a sugar control, EI was reduced in groups ingesting saccharin, Asp, cyclamate, sucralose, Ace-K, stevia, and erythritol, and it may have also been reduced in comparison with water control groups (5, 9–11).

The metabolic inertness of NNSs in the gastrointestinal tract is another debated topic. In vitro studies using isolated cells and animal experiments have suggested that NNSs activate T1R2/T1R3 in the small intestinal epithelium. This increases glucose transport across the gut *1*) in the short term via the insertion of the facilitative glucose transporter GLUT2 into the apical membrane of enterocytes (12) and *2*) in the long term via release of glucagon-like peptide 1 (GLP-1) and glucose-dependent insulinotropic peptide (GIP) from enteroendocrine cells, resulting in increased expression of the sodium-dependent glucose transporter SGLT1, mRNA, and protein in enterocytes (13, 14). Such studies suggest that NNSs may alter postprandial blood glucose and incretin levels and thus affect appetite. Evidence from human studies are not as clear, and meta-analyses found that NNSs such as Asp, saccharin, stevioside, and sucralose, when given alone or with a nutrient preload, do not cause a change in short-term blood glucose (15) and insulin levels (16). A systematic review (17) reported that 75% of the studies found no significant gut peptide response after NNS intake.

Of the systematic reviews (5, 10, 11, 15–19) evaluating the effects of NNSs on appetite measures, a commonly mentioned source of heterogeneity is the different types of NNSs used in the studies. Moreover, the systematic reviews generalize the effects found in the body to all NNSs since there is currently no clinical evidence that the metabolic effects of NNSs differ. However, the biological fates of ingested NNSs do differ (4): Asp is rapidly broken down in the small intestine to aspartic acid, phenylalanine, and methanol, whereas Ace-K remains intact and is absorbed across the gut and excreted by the kidney. Ace-K (but not Asp) therefore potentially interacts with sweet taste receptors expressed along the length of the gut as well as in other tissues, such as the hypothalamus and brain stem (20). Last, very few of the review studies investigated the metabolic effects of an Asp/Ace-K blend. Asp and Ace-K are the most popular NNSs consumed globally and are often found blended in foods and beverages—this serves to mask the bitter aftertaste of Ace-K (13) and to better emulate the taste and texture of sucrose-sweetened products (14). Therefore, our aim was to compare the effects of Asp/Ace-K blends with sugar or water on appetite and avoid the potential confounding effects of other NNSs by *1*) including studies investigating only Asp/Ace-K blends and *2*) conducting a meta-analysis on multiple factors contributing to appetite: EI, subjective appetite, and postprandial blood glucose and incretin hormones.

## Methodology

A systematic review was undertaken to identify randomized controlled trials (RCTs) in humans that investigated the effects of the NNSs Asp and Ace-K (Asp/Ace-K) on EI, subjective appetite levels, and postprandial blood glucose and the incretins GLP-1 and GIP. A study protocol was prepared in line with the *Cochrane Handbook for Systematic Reviews of Interventions* (21) and PRISMA guidelines (Preferred Reporting Items for Systematic Reviews and Meta-Analyses) (22) and registered with the International Prospective Register of Systematic Reviews (PROSPERO: CRD42017061015).

The databases MEDLINE, Web of Science, and Cochrane CENTRAL databases (Embase, PubMed, and CINAHL) were searched from all years of record until 17 May 2021 with no restrictions on language. A combination of MeSH terms and keywords, such as “aspartame” AND “food intake” OR “blood glucose,” were used to identify relevant studies. The complete search strategy is shown in **Supplemental Table 1**. We also searched the reference lists of the studies included in the systematic review and the clinicaltrials.gov register, but this yielded no additional peer-reviewed data sets for analysis.

### Deviations from registered protocol

Our initial search strategy protocol included RCTs using Asp and Ace-K alone as well in combination, but in our final analysis we focused only on studies that analyzed the effects of Asp/Ace-K blends, since they are commonly used in foods and beverages. In our original search strategy, we also included weight change, BMI, body composition outcomes, and appetite-controlling peptides, such as cholecystokinin (CCK) and ghrelin, but these were not included in our final analysis as there were not enough data to form a systematic review.

### Study inclusion and exclusion criteria

Full details of the inclusion and exclusion criteria are shown in **Table 1**. Studies containing sucralose, erythritol, cyclamate, and saccharin in minor amounts (<20% NNS dose) were also included. RCTs in individuals with normal weight (NW), overweight, and obesity and those with type 2 diabetes mellitus (T2DM) were included, whereas those with other chronic diseases were excluded, such as type 1 diabetes. The glucose homeostasis abnormalities associated with T2DM are well recognized, but the impact of Asp/Ace-K blends in other chronic illnesses may have presented confounding effects and so was excluded. Non-RCTs and observational studies were excluded to minimize biases associated with these study designs and because combining study designs may have caused the results to be weighted toward observational study estimates due to the higher sample sizes as compared with RCTs (23).

**TABLE 1:** PICOS inclusion and exclusion criteria of studies in the systematic review and meta-analysis of the effects of Asp/Ace-K blends on human appetite, blood glucose, and incretins^1^

PICOS	Inclusion	Exclusion
Population	• Male and females, humans• Normal weight, overweight, obese^2^• >18 y old• Type 2 diabetes mellitus	• Animals/cells• <18 y old• Other diagnosed chronic diseases
Intervention	Foods/beverages sweetened with Asp and Ace-K Asp and Ace-K and other NNSs	Foods/beverages sweetened without Asp and Ace-K together
Comparisons	Foods/beverages with Water Sucrose Glucose	
Outcomes	• Energy intake• Subjective appetite• Blood glucose• GLP-1• GIP	
Study design	• Randomized controlled trials• Laboratory/free-living• Acute (<1 d)• Chronic (>1 d)	• Nonrandomized controlled trials• Observational/cohort studies

1 Ace-K, acesulfame-K; Asp, aspartame; GIP, glucose-dependent insulinotropic peptide; GLP-1, glucagon-like peptide 1; NNS, nonnutritive sweetener.

2 As defined by the WHO guidelines (58).

### Data extraction

Titles and abstracts of the identified studies were independently screened by 3 reviewers (KM, YC, and CPC) against the inclusion and exclusion criteria. KM and YC retrieved the full-text articles for the studies deemed eligible for further scrutiny. If KM and YC were unsure regarding study eligibility, clarification was sought from CPC.

Design of the data extraction form was based on guidelines from Cochrane (21) and included first author's name and year, number of participants, gender and BMI range, study length, setting (free-living or laboratory), and methodology, including the type and amount of NNS and comparator used and the outcomes assessed.

### Data extraction for meta-analysis

Mean or mean difference (MD) and SD or SEM for EI for the Asp/Ace-K blends and control groups were extracted or calculated and synthesized into the meta-analysis. For blood glucose changes, peak blood glucose (mmol/L) at 15–30 min was analyzed, rather than AUC, as it was the only blood glucose parameter that could be reliably extracted from the included studies. If means and SD or SEM were not reported in text or tables for any outcome, the corresponding author was contacted for clarification by email (maximum 3 attempts made). If data were still unavailable but shown in a graph, they were extracted using a computer visual screen ruler application. Visual analog scale data for hunger, desire to eat, feelings of fullness and alertness (24), and incretins were summarized descriptively.

### Risk of bias

The risk of bias of each study was assessed using the guidelines from the Cochrane handbook (25). Selection, performance, detection, attrition, and other causes of bias, such as that due to industry support or collaboration, were judged by KM and YC as having a high, low, or unclear risk.

### Statistical analysis

Studies were grouped according to outcomes measured: EI, blood glucose, GLP-1, and GIP. Subgroup analyses were carried out when possible (≥2 studies) according to the comparator used [sugar (sucrose, glucose) or water] and study length (acute: <1 d; chronic: >1 d).

Statistical analyses were performed using Review Manager 5.4.1 (Cochrane). The raw mean difference was calculated; the inverse variance method was used to weight the studies; and data were pooled using a random effects model to quantify differences in means. *P* < 0.05 was considered statistically significant.

Between-study heterogeneity was tested and quantified using the *I*^2^ statistic. According to the Cochrane handbook, an *I*^2^ value of 0%–40% might not be important, 30%–60% may represent moderate heterogeneity, 50%–90% may represent substantial heterogeneity, and 75%–100% may represent considerable heterogeneity (21). Publication bias can be assessed by visual inspection of funnel plots if ≥6 studies are available for analysis (26).

## Method of Incorporating Crossover Trials into Meta-Analysis

The studies in this review were a mixture of parallel and crossover study designs. The data required for a meta-analysis for parallel study designs were the mean or MD and SD or SEM and were included in all parallel studies in this review. The data required for a meta-analysis for crossover study designs, including SE of the mean difference (SE_MD_) or SD of the difference (SD_Diff_), were not included for most crossover trials in this review. To incorporate crossover studies with missing data into the meta-analysis, the Cochrane guidelines describe 3 possible approaches in section 16.4.5 (21). One approach, however, involved including data from only the first period of each trial, which was not possible due to how the trials in our study were designed. The first approach that we implemented was to input the data as if the trial were a parallel study design, using the absolute data; the second approach was to impute the missing SE_MD_ and SD_Diff_ by using a correlation coefficient. We used the following calculation to determine SD_Diff_:

(1) $$\begin{equation*}
{{\rm SD}_{\rm Diff}} = \surd \left( {\rm SD}_{\rm E}^{\rm 2} + {\rm SD}_{\rm C}^{\rm 2} - ({\rm 2 \,\, \times Corr}\,\, {\times}\,\, {\rm SD}_{\rm E}\,\,{\rm \times}\,\, {\rm SD}_{\rm C}) \right)
\end{equation*}$$

where E = experimental condition (Asp/Ace-K), C = control condition, and Corr = correlation coefficient. For the latter, we assigned a maximum value of 0.63 for missing Corr values based on an average of the calculated Corr values and from previous literature (9). SE_MD_ was then determined by dividing SD_Diff_ by the square root of the study sample size (*N*):

(2) $$\begin{equation*}
{{\rm SE}_{\rm MD}} = {{\rm SD}_{\rm Diff}}\,\,/\,\,\surd N
\end{equation*}$$

A sensitivity analysis was conducted for the EI and blood glucose outcomes by comparing preliminary effect sizes from the meta-analysis results by using only absolute data as reported in studies or by combining studies with imputed values as described earlier. The method resulting in the most conservative effect size was then utilized for all outcomes of interest.

## Results

### Literature search

**FIGURE 1:**
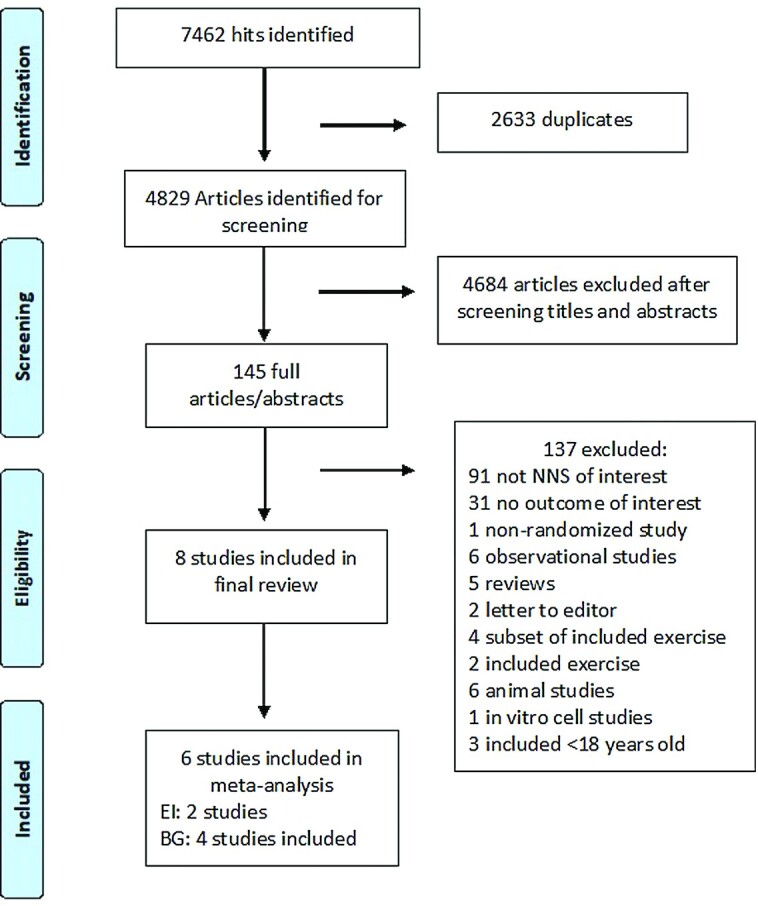
PRISMA diagram of literature search, screening, and selection of articles for inclusion in this systematic review on the effect of aspartame/acesulfame-K blends on human energy intake (EI), subjective appetite, blood glucose (BG), glucagon-like peptide 1, and glucose insulinotropic peptide. NNS, nonnutritive sweetener; PRISMA, Preferred Reporting Items for Systematic Reviews and Meta-Analyses.

The details of the literature search are presented in **Figure 1**. Once duplicates were removed, 4829 publications were identified for screening, and from this, 145 were retrieved in complete text for full review. Four studies (27–30) were identified that used Asp/Ace-K blends, but those studies also contained sucralose, erythritol, cyclamate, and saccharin in amounts > 20% NNS dose or did not specify the doses used and so were excluded from further analysis. Another study was excluded because the study participants were not randomly assigned (31). Eight studies were identified as appropriate for inclusion, and their characteristics are presented in **Table 2**.

**TABLE 2:** Characteristics of studies that evaluated the effects of Asp/Ace-K blends on human energy intake, subjective appetite, blood glucose, GLP-1, and GIP when compared with sugar or water controls^1^

Author (year)	Population^2^	Length	Methodology	Outcomes
Bonnet et al. (2018) (35)	*n* = 5028 F, 22 M28 NW, 22 O	Chronic	Crossover, free-living, 2 × 12-wk intervention period with 4-wk washout period in between, drink 2×/d: A) 330 mL carbonated water B) 330 mL carbonated water with129 mg Asp and 13 mg Ace-K	• Energy intake per 24 h
Holt et al. (2000) (36)	*n* = 11MNW	Acute	Crossover, laboratory session; after consuming standardized breakfast at home, subjects arrived and consumed preload of 375 mL of 1 of the following: A) Plain carbonated mineral water B) Sucrose soft drink (*Coca-Cola*®) C) Sugar-free soft drink (Asp/Ace-K*Coca-Cola*®)110 min to ad libitum lunch	• Energy intake per meal• Subjective appetite:hunger, fullness
Kim et al. (2020) (34)	*n* = 3926 F, 13 MNW, O, Ob	Chronic	Crossover, free-living, 2 wk, subjects allocated to consume 0.6 L/d of either A) Mineral water B) Artificially sweetened beveragewith Asp (144 mg/L) and Ace-K(211 mg/L)	• Blood glucosemeasured over 60 min
Olalde-Mendoza and Moreno-Gonzalez (2013) (39)	*n* = 8063 F, 17 MO, Ob^3^	Acute	Parallel, laboratory sessions, subjects allocated to either A) *n* = 40 had sucrose soft drink B) *n* = 40 diet Asp/Ace-K beverage	• Blood glucosemeasured over 30 min
Panahi et al. (2013) (32)	*n* = 2914F, 15 MNW	Acute	Crossover, laboratory sessions, 1-wk washout period; after consuming standardized breakfast at home, subjects given ad libitum intake of A) Water (control) B) Regular cola (sucrose) C) Diet cola (Asp/Ace-K) With ad libitum lunch	• Energy intake per meal• Subjective appetite:hunger, fullness, desireto eat• Blood glucosemeasured over 120 min
Solomi et al. (2019) (38)	*N* = 106F, 4 MNW	Acute	Crossover, laboratory session, subjects arrived fasted and given 1 of the following: A) 25 g glucose in 125 mL water+ 236 mL water B) 25 g glucose in 125 mL water+ 236 mL diet cola (Asp/Ace-K) C) 125 mL water + 236 mL cola (sucrose)	• Blood glucosemeasured over 120 min
Sylvetsky et al. (2016) (33)	*n* = 3117F, 14 MNW, O, Ob	Acute	Crossover, laboratory session, at least 1-wk washout period, subjects arrived fasted and given either A) 355 mL seltzer water B) 355 mL caffeine-free Diet MountainDew® (57 mg Asp, 18 mg Ace-K, 18 mgsucralose) Prior to an oral glucose tolerance test	• Blood glucose• GLP-1• GIP• Measured over 130 min
Van Wymelbeke et al. (2004) (37)	*n* = 2412F, 12 MNW	Chronic	Crossover, free-living, 10 wk, subjects alternated mineral water sweetened with either A) Sucrose with orange or raspberry flavor B) Asp (20 mg/L), Ace-K (110 mg/L),saccharin (30 mg/L)	• Energy intake per 48 h• Subjective appetite:hunger

1 Ace-K, acesulfame-K; Asp, aspartame; GIP, glucose-dependent insulinotropic peptide; GLP-1, glucagon-like peptide 1.

2 Sample size, sex, and weight status [normal weight (NW), obese (Ob), overweight (O)].

3 Includes type 2 diabetes.

### Design

The 8 studies in the review consisted of 274 participants—of which 166 were females and 108 were males; their weight status ranged from NW to overweight and obese (Table 2). Five studies were acute interventions (<1 d) and 3 were chronic (2–12 wk); similarly, 1 study was a parallel study design and 7 were crossover. All studies used a blend of Asp and Ace-K as experimental conditions, with a sugar or water control. Four studies measured EI; 3 studies, subjective appetite scores; 4 studies, postprandial blood glucose; and 1 study, GLP-1 and GIP.

### Risk of bias analysis

**FIGURE 2:**
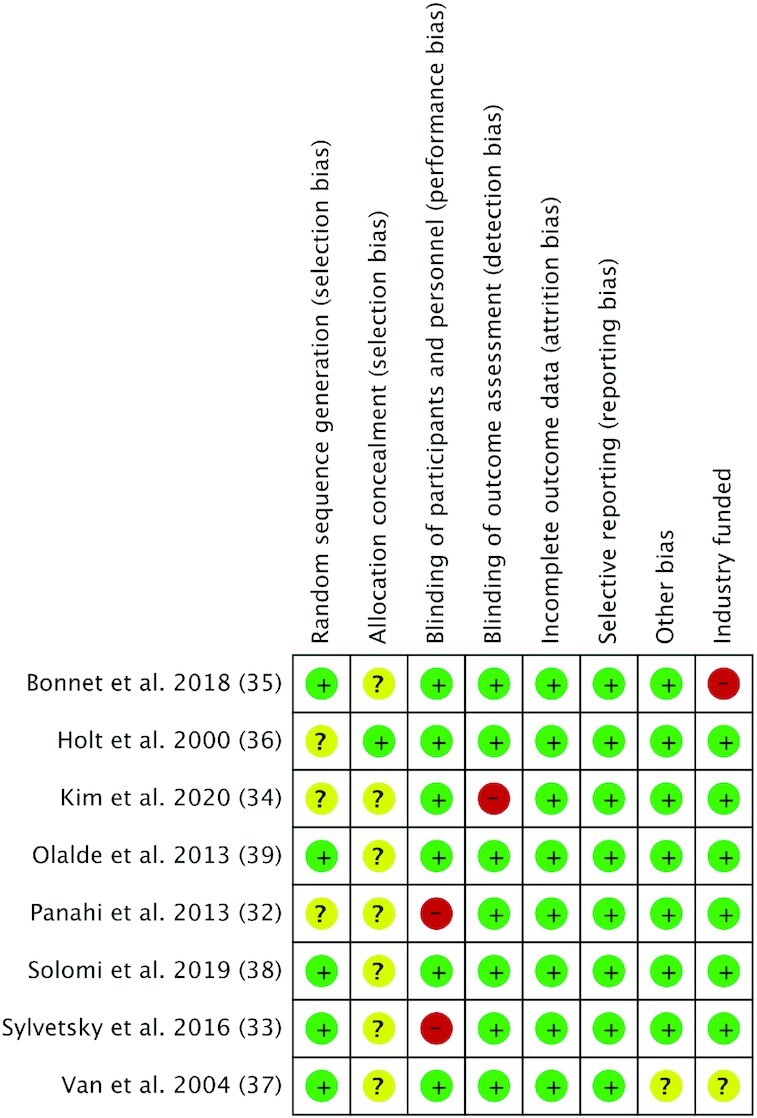
Risk of bias analysis of included articles that assessed the effects of aspartame/acesulfame-K blends on human appetite, blood glucose, and incretins. Green, low risk; yellow, unclear risk; red, high risk.

The risk of bias of each study is presented in **Figure 2**. Most studies were at a low risk for performance bias, except for 3 (32–34) that were at a high risk, as blinding of participants, personnel, or health outcomes was not mentioned. One study (35) was at high risk of bias due to industry support or collaboration, as it was funded by an organization or company associated with sugar or NNS products.

A summary of the key findings for each outcome and NNS is presented in **Table 3**.

**TABLE 3:** Key findings evaluating the effects of Asp/Ace-K blends on human energy intake, appetite, blood glucose, and incretins when compared with sugar or water controls^1^

Outcomes	Asp/Ace-K vs. controls
Energy intake	• Asp/Ace-K < water (*P* = 0.002)
	• Asp/Ace-K < sugar (*P* = 0.004)
Subjective appetite	• 1 of 3 studies significantly found:Asp/Ace-K < water
	• 2 of 3 studies no difference between groups
Blood glucose	• No significant difference between Asp/Ace-K when compared with water and sugar controls
GLP-1	• No significant difference between Asp/Ace-K when compared with water and sugar controls
GIP	• No significant difference between Asp/Ace-K when compared with water and sugar controls

1 Ace-K, acesulfame-K; Asp, aspartame; GIP, glucose-dependent insulinotropic peptide; GLP-1, glucagon-like peptide 1.

### Energy intake

**FIGURE 3:**
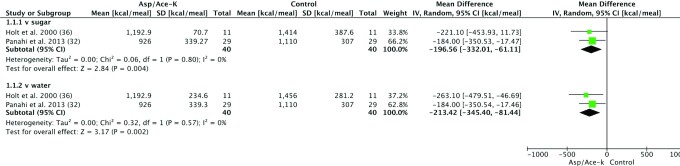
Forest plot of the difference in energy intake (kcal/d) in human participants after aspartame/acesulfame-K (Asp/Ace-K) intake compared with sugar or water controls. Study length ranged from 1 d to 24 wk.

Four studies assessed EI after Asp/Ace-K intake and included 114 participants (**Figure 3**). The overall effect for Asp/Ace-K blends compared with sugar showed a significantly reduced EI in the groups receiving Asp/Ace-K (MD: –196.56 kcal/meal; 95% CI: –332.01, –61.11 kcal/meal; *P* = 0.004) with low heterogeneity (*I*^2^ = 0%, *P* = 0.8). EI was also significantly reduced in the groups receiving Asp/Ace-K blends when compared with water controls (MD: –213.42 kcal/meal; 95% CI: –345.40, –81.44 kcal/meal; *P* = 0.002) with low heterogeneity (*I*^2^ = 0%, *P* = 0.57).

A sensitivity analysis was undertaken to determine if effect sizes differed if the meta-analysis was conducted using imputed values for crossover studies with missing data, instead of absolute values (see **Supplemental Figure 1**). When using imputed values for Asp/Ace-K blends compared with sugar, effect sizes were slightly smaller (MD: –193.04 kcal/meal; 95% CI: –279.12, –106.95 kcal/meal) but still statistically significant (*P* < 0.0001) with low heterogeneity (*I*^2^ = 0%, *P* = 0.72). For Asp/Ace-K compared with water, the effect size was also reduced (MD: –145.18 kcal/meal; 95% CI: –367.57, 77.21 kcal/meal), which was not significant (*P* = 0.2) with high heterogeneity (*I*^2^ = 87%, *P* = 0.006).

Only 4 studies could be included in the meta-analysis of EI, and so assessment of publication bias by funnel plot was not meaningful.

### Subjective appetite scores

Three studies measured subjective appetite ratings comparing Asp/Ace-K intake with water (32, 36) or sugar (32, 36, 37), with 2 studies (32, 36) reporting data on both comparisons (**Table 4**). An overall 64 NW subjects were included. One study provided data in extractable graphs (32), and the 2 other studies provided hunger and fullness data in tables (36, 37)

**TABLE 4:** Results of studies assessing the effects of Asp/Ace-K blends on human subjective appetite scores when compared with sugar and water controls^1^

Comparison: Author (year)	Appetite measured	Results
Asp/Ace-K vs. water		
Holt et al. (2000) (36)^2^	Hunger, fullness	NS
Panahi et al. (2013) (32)^2^	Hunger, fullness, desire to eat	Appetite^3^: ↓ (*P* < 0.05)
Asp/Ace-K vs. sugar		
Holt et al. (2000) (36)^2^	Hunger, fullness	NS
Panahi et al. (2013) (32)^2^	Hunger, fullness, desire to eat	Appetite^3^: NS
Van Wymelbeke et al. (2004) (37)^4^	Hunger	NS

1 Ace-K, acesulfame-K; Asp, aspartame.

2 Study length < 1 d.

3 Appetite: hunger, fullness and desire to eat pooled.

4 Study length > 1 d.

There was little consistency between studies in appetite variables measured (hunger, desire to eat, fullness, and satiety); therefore, data were summarized as a narrative. Two of the 3 studies reported no significant differences between the Asp/Ace-K group and the control group in any of the appetite variables measured. Panahi et al. (32) pooled the ratings for hunger, desire, and fullness into 1 “appetite” variable and reported the Asp/Ace-K group to have significantly lower appetite 30–120 min postintake and significantly lower AUC compared with the water control but with no difference against the sugar group.

### Blood glucose

**FIGURE 4:**
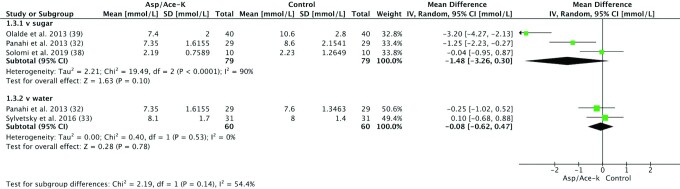
Forest plot of the difference in blood glucose peak concentration (mmol/L) in human participants 15–30 min after aspartame/acesulfame-K (Asp/Ace-K) intake compared with sugar and water controls. Study length ranged from 1 d to 10 wk.

Four of 5 studies that measured postprandial blood glucose provided sufficient data to be included in a meta-analysis (**Figure 4**). Data from 1 study (32) was extracted from graphs; 2 studies (33, 38) provided data in tables; and one study (39) provided SD of the mean change upon email request. Peak concentration (Cmax) of blood glucose 15–30 min after Asp/Ace-K intake was compared with water or sugar control and included 150 subjects of mostly NW—although Olalde-Mendoza and Moreno-Gonzalez (39) included participants with T2DM and Sylvetsky et al. (33) included participants who were overweight and obese.

Subgroup analysis according to type of comparison—sugar or water—showed a nonsignificant reduction in blood glucose in groups receiving Asp/Ace-K blends compared with sugar (MD: –1.48 mmol/L, 95% CI: –3.26, 0.3 mmol/L; *P* = 0.1) with substantial heterogeneity (*I*^2^ = 90%, *P* < 0.0001) and no difference between Asp/Ace-K blends and water (MD: –0.08 mmol/L; 95% CI: –0.62, 0.47 mmol/L; *P* = 0.78) with low heterogeneity (*I*^2^ = 0%, *P* = 0.53) (Figure 4).

A sensitivity analysis was done to compare if effect sizes differed if meta-analysis was conducted using imputed values for crossover studies with missing data, instead of the absolute values previously shown. Based on imputed values, MD, effect size, and heterogeneity were similar for the sugar subgroup [MD: –1.42 mmol/L (95% CI: –2.96, 0.11 mmol/L), *P* = 0.07; *I*^2^ = 94%, *P* < 0.00001] and for the water subgroup [MD: –0.06 mmol/L (95% CI: –0.40, 0.28 mmol/L), *P* = 0.72; *I*^2^ = 13%, *P* = 0.28) (**Supplemental Figure 2**).

### Incretins

There was an insufficient number of studies to undertake a meta-analysis of GIP and GLP-1 responses to Asp/Ace-K blends. Sylvetsky et al. (33) compared an Asp/Ace-K blend with water controls and found no difference in the blood levels of GLP-1 or GIP.

## Discussion

In this systematic review and meta-analysis, we found that in individuals who were healthy, EI was significantly reduced after consuming a blend of Asp/Ace-K compared with sugar and water controls; however, the data were insufficient to establish if the reduction in EI was linked to subjective appetite or changes in postprandial blood glucose or the incretins GLP-1 and GIP.

Our results show that EI was significantly reduced following Asp/Ace-K intake compared with sucrose beverages/foods with low heterogeneity between the studies analyzed. Wiebe et al. (9) and Rogers et al. (5) came to similar conclusions in regard to sucrose compared with NNSs in their systematic reviews and meta-analysis, although both their reviews observed high heterogeneity, which was likely due to differences in study design, population, weight status, and dosage/administration of test beverages/foods. Indeed, Wiebe et al. included studies using Asp alone, and Rogers et al. performed a meta-analysis on short-term (acute) intervention studies and did not exclude studies based on NNS type. In the acute studies (<1 d), calories from the test preloads were not included, so EI was reduced due to reduced consumption posttreatment; in the larger review (5), calories from the sucrose preloads were adjusted for. For the long-term interventions (2–10 wk), it is possible that the reduction in caloric intake in the Asp/Ace-K group compared with sucrose controls was due to the NNS group replacing sugar-sweetened beverage calories with noncaloric NNS beverages. Indeed, from the articles included in our analysis, there was no evidence of EI compensation, either full or partial, by other foods and beverages in the diet following intake of Asp/Ace-K blends. Therefore, it seems likely that in people who are healthy at least, NNSs can promote a satiety effect—that is, a feeling of fullness between meals. Compensatory mechanisms may, however, differ in individuals who are diabetic or obese. Indeed, observational studies have shown a positive association between NNS intake and obesity (40). Although this may be due to reverse causality and even though the preponderance of evidence from interventional studies suggests that NNSs can result in lower EI when compared with controls, future interventional studies on the effects of NNSs in individuals who are diabetic or obese are needed.

EI was also significantly reduced following Asp/Ace-K intake when compared with water controls with low heterogeneity between the studies analyzed; yet, when we ran a sensitivity analysis, the decrease in EI was no longer significant (*P* = 0.2) with high heterogeneity, similar to the findings by Rogers et al. (5). The number of comparisons in our subgroup (*n* = 2) compared with theirs (*n* = 35) may have influenced our initial results, and it should be noted that Rogers et al. included studies with other NNSs, which may be a confounding factor. Moreover, in 2 large systematic reviews (5, 41), body weight was significantly reduced in the NNS groups compared with the sucrose and water controls, providing further evidence for chronic intake of NNS influencing EI.

The majority of studies reported no significant differences in hunger, fullness, satiety, or desire-to-eat scores between the Asp/Ace-K and control interventions; therefore, it is unclear whether the reduction in EI seen in the Asp/Ace-K group can be linked to the subjective appetite scores. However, of the studies that did report significantly higher hunger and lower appetite ratings in the Asp/Ace-K group, EI was correspondingly higher and lower (32). Although the use of a visual analog scale to rate appetite has been validated and seen to be an effective method especially in crossover and laboratory experiments (24), differences in parallel study designs or timing of meals and in test meal/beverage administration may contribute to our inconclusive summary. Panahi et al. (32) observed significantly lower appetite in the Asp/Ace-K group compared with water control, but subjects in the study were given test beverages ad libitum. The volume of beverage consumed has been illustrated to affect satiety and EI (42) and therefore may influence appetite. Asp and sucralose have been linked to higher satiety compared with water and sucrose (43, 44), but similar to the studies in this review, many studies have not shown an effect (45–47).

Our meta-analysis findings suggest that Asp/Ace-K blends do not affect blood glucose levels. Asp/Ace-K blends had no significant effect on Cmax when compared with water, which does not raise blood glucose levels. Asp/Ace-K blends also had a reduced effect on blood glucose when compared with sucrose, which does raise blood glucose levels; this may again be interpreted as a lack of an effect on blood glucose levels by Asp/Ace-K. The lower Cmax in the Asp/Ace-K groups when compared with sugar controls, however, did not achieve statistical significance (*P* = 0.1–0.07) possibly because so few studies using Asp/Ace-K blends have been undertaken.

To our knowledge, a meta-analysis comparing blood glucose Cmax of Asp/Ace-K blends with controls has not been conducted before. Nichol et al. (15) undertook a meta-analysis on postprandial blood glucose from studies investigating the NNSs: Asp, saccharin, sucralose, and stevia, ingested on their own, generating an estimated trajectory of blood glucose from time of intake to 210 min postintake. In support of our findings, they reported that these NNSs did not cause an increase in blood glucose levels from baseline but gradually declined over the 210 min observation; the last interval (180–210 min) was significantly lower than baseline, but this was found to have significant publication bias.

Other systematic reviews that did not conduct meta-analyses (17–19) offer further support to our findings and have narratively summarized that >75% of the studies investigating NNSs ingested alone or with a carbohydrate-containing meal or beverage did not affect blood glucose concentrations. However, the aforementioned reviews did not include studies using Asp/Ace-K blends, with a few exceptions. Tucker and Tan (19) included in their review the study by Sylvetsky et al. (33), which showed no significant differences in blood glucose between NNSs and the water control. In addition, Romo-Romo et al. (17) included in their review the study by Olalde-Mendoza and Moreno-Gonzalez (39), who found no significant differences in blood glucose levels in individuals consuming the Asp/Ace-K blend when compared with a sugar-sweetened beverage; yet, their study did show differences in blood glucose levels between groups over a 30-min period. Finally, in this report, we show that blood glucose Cmax had the highest MD between the Asp/Ace-K blend and the sucrose control (–3.2 mmol/L; 95% CI: –4.27, –2.13 mmol/L).

Conflicting findings on the effects of NNSs on blood glucose are most likely due to differences in the study population, the method of NNS administration, and how the effects on blood glucose were measured. Indeed, the high heterogeneity observed in Figure 4 may have been due to the fact that the study by Olalde-Mendoza and Moreno-Gonzalez (39) included participants with T2DM unlike the rest of the studies, which had participants who were healthy. Post hoc removal of their study from the analysis reduced the sugar subgroup heterogeneity from *I*^2^ = 90% (*P* < 0.0001) to *I*^2^ = 68% (*P* = 0.08), and Cmax in the Asp/Ace-K group compared with the sugar subgroup was not significantly different (MD: –0.63 mmol/L; 95% CI: –1.82, 0.55 mmol/L; *P* = 0.3; see **Supplemental Figure 3**), suggesting that Asp/Ace-K blends and sucrose may have similar effects on blood glucose Cmax in healthy individuals but not in those with T2DM. In addition, Sakurai et al. (29) combined sucrose with Asp/Ace-K and reported a lower MD (–0.7 mmol/L; 95% CI: –1.58, 0.18 mmol/L), suggesting that an antagonistic effect may occur between sucrose and Asp/Ace-K blends, which is contrary to the synergistic effects reported in rodent studies (12, 14). Finally, in the systematic review and network meta-analysis undertaken by Wiebe et al. (9), 3 studies were identified consisting of participants who were healthy: 2 compared Asp and sucrose, and 1 compared sucralose and fructose. When assessing the MD between overnight fasting blood glucose and 2-h blood glucose post–NNS treatment, the authors reported no significant differences between groups, but AUC, Tmax (time to reach peak concentration), and Cmax were not reported.

Although our results combined with the cited systematic reviews and meta-analyses suggest that there might be little or no difference between types of NNS and blood glucose responses, more short- and long-term studies measuring the effects of NNS on Cmax, Tmax, and 2- to 3-h AUC responses are needed in individuals who are healthy or have T2DM or obesity. Postprandial glycemic dips have also been shown to be an accurate predictor of appetite and EI in healthy individuals (48), and so blood glucose changes during the return-to-baseline phase should be investigated.

A small number of studies measuring GLP-1 and GIP after Asp/Ace-K intake have been undertaken (29, 30, 33), but only the Sylvetsky et al. (33) study was included in this review and no significant difference between interventions was observed. No studies were identified in our original search strategy that measured the effects of Asp/Ace-K blends on ghrelin, CCK, and other gut peptides, and no meta-analysis has been done previously. Yet, a systematic review consisting of studies measuring incretin hormones as well as other appetite-regulating hormones, such as insulin, glucagon, peptide YY, and CCK, among others, found that out of 20 studies measuring these outcomes, only 5 reported any significant effects in GLP-1 and insulin in response to sucralose and Asp (17). In vitro data indicate that NNSs may stimulate incretin secretion from enteroendocrine cells (13, 14), which would stimulate insulin synthesis and secretion directly, lowering blood glucose and ghrelin levels and affecting satiety, or effects of NNS may be indirect via an increase in small intestinal glucose uptake and blood glucose (49, 50), although the findings in humans are conflicting (46, 51–56). It is therefore unclear whether intake of Asp/Ace-K blends affects gut peptide and insulin release, and so more studies in humans are required.

### Strengths and limitations

Strengths of this review include the following: a protocol was published prior to starting the review; a comprehensive and systematic search was conducted to identify relevant studies without any search or language restriction; and the review was executed in line with the PRISMA reporting guidelines. Additionally, 3 independent reviewers screened the abstracts identified, and support from an independent expert in statistics was given to clarify how to best input the data for the meta-analysis. Our EI meta-analysis was limited by the numbers of studies that we could include because investigators did not report EI consistently, with some expressing EI per meal and others expressing their data as EI per day or 48 h. Another limitation includes the inability to extract data from the majority of studies reporting subjective appetite ratings due to a lack of numerical data and inconsistencies in the appetite variables that were measured; thus, no meta-analysis was performed. This limitation may highlight the need for authors to present subjective appetite results in a more useful manner to better understand how appetite ratings can be linked to physiologic findings. Similarly, the parameters used to assess changes in blood glucose levels between groups were inconsistent. Another limitation pertains to our inclusion of outcome measures: studies measuring body weight and other appetite-regulating hormones or factors, such as insulin, glucagon, leptin, ghrelin, CCK, and gastric emptying, were not included, and these factors affect appetite as well and should be considered in future meta-analyses. Studies in this review needed to be clearer on methods of randomization and allocation concealment, but other biases, such as blinding, dropout rates, and selective reporting of data, were not an issue. Industry bias due to its support or collaboration may have been present in our analysis, although we did not find that studies were at an especially high risk of bias. Industry support could conceivably influence study design, outcomes studied, or the decision to publish. It therefore remains important that potential conflicts of interest be openly declared in all publications. High heterogeneity in some of our meta-analysis suggests that caution is needed when drawing conclusions from studies that may not have been similar enough to combine. In the reviewed studies, there was also wide variation in the Asp/Ace-K doses and formulations. Some studies used commercially available diet drinks containing an Asp/Ace-K blend (32, 33, 36, 38), whereas others used a mixture of Asp (20–390 mg/L) and Ace-K (39–211 mg/L) at ratios of 1:10 (35) 1:0.7 (34) 1:0.2 (37), and 1:0.3 (33) and some contained additional sweeteners at relatively low levels, such as sucralose (50 mg/L) and saccharin (30 mg/L). Commercial blends of Asp/Ace-K vary and are in the range of 1:0.5–0.25. Future studies on the effects of NNSs should only use doses and formulations that are dietarily relevant. Last, there was evidence of small study publication bias in the EI analysis, which may be present in the other analyses; future systematic reviews should aim to search for and include more unpublished or non-English studies.

### Conclusion

In conclusion, our findings are in line with previously published systematic reviews (5, 9) showing that NNS intake can lead to a reduction in EI compared with caloric (sugar) or noncaloric (water) intake. We could not confirm whether the reduction in EI was associated with subjective appetite ratings or incretin hormones, but it is a possibility that the slight decrease in blood glucose in the Asp/Ace-K group compared with sugar may be linked to the decrease in food intake—although this would be opposite to evidence seen in humans consuming carbohydrates (57), where an inverse relationship has been shown between blood glucose levels and subsequent food intake and subjective appetite scores. In addition, the small number of studies and, in some cases, high heterogeneity make it difficult to come to a singular conclusion regarding the effects of Asp/Ace-K blends on appetite and associated biomarkers. More studies in individuals who are healthy or have T2DM or obesity are required, as well as studies using products that have reduced added sugar content but contain NNS to maintain palatability.

## Supplementary Material

nmac072_Supplemental_Files

## Notes

The authors reported no funding received for this study.

Author disclosures: The authors report no conflicts of interest.

Supplemental Figures 1–3 and Supplemental Table 1 are available from the “Supplementary data” link in the online posting of the article and from the same link in the online table of contents at https://academic.oup.com/advances/.

Abbreviations used: Ace-K, acesulfame-K; Asp, aspartame; CCK, cholecystokinin; Cmax, peak glcuose concentration; EI, energy intake; GIP, glucose-dependent insulinotropic peptide; GLP-1, glucagon-like peptide 1; MD, mean difference; NNS, nonnutritive sweetener; NW, normal weight; RCT, randomized controlled trial; T2DM, type 2 diabetes mellitus.
