# Supplementary material for: The Combined Effects of Aspartame and Acesulfame-K Blends on Appetite: A Systematic Review and Meta-Analysis of Randomized Clinical Trials
Source: Adv Nutr. 2022 Sep 3;13(6):2329–40. doi: 10.1093/advances/nmac072 (PMC9776645; doi:10.1093/advances/nmac072)
Supplement: nmac072_Supplemental_Files [file nmac072_supplemental_files.zip › merge_3_supplementary_data_files_CORRECTED.pdf]

The combined effects of Aspartame and Acesulfame-k blends on appetite: a systematic review and meta-analysis of randomised clinical trials

Kirnjot Mehat

Online supplementary material

Supplemental figure 1

Study or Subgroup	Mean Difference	SE	Weight	Mean Difference IV, Random, 95% CI
**1.6.1 sugar**				
Holt et al. 2000 (36)	−221.1	89	24.4%	−221.10 [−395.54, −46.66]
Panahi et al. 2013 (32)	−184	50.5	75.6%	−184.00 [−282.98, −85.02]
**Subtotal (95% CI)**			**100.0%**	**−193.04 [−279.12, −106.95]**
Heterogeneity: Tau² = 0.00; Chi² = 0.13, df = 1 (P = 0.72); I² = 0%				
Test for overall effect: Z = 4.39 (P < 0.0001)				
**1.6.2 water**				
Holt et al. 2000 (36)	−263.1	66.3	48.1%	−263.10 [−393.05, −133.15]
Panahi et al. 2013 (32)	−36	49.1	51.9%	−36.00 [−132.23, 60.23]
**Subtotal (95% CI)**			**100.0%**	**−145.18 [−367.57, 77.21]**
Heterogeneity: Tau² = 22383.95; Chi² = 7.58, df = 1 (P = 0.006); I² = 87%				
Test for overall effect: Z = 1.28 (P = 0.20)				

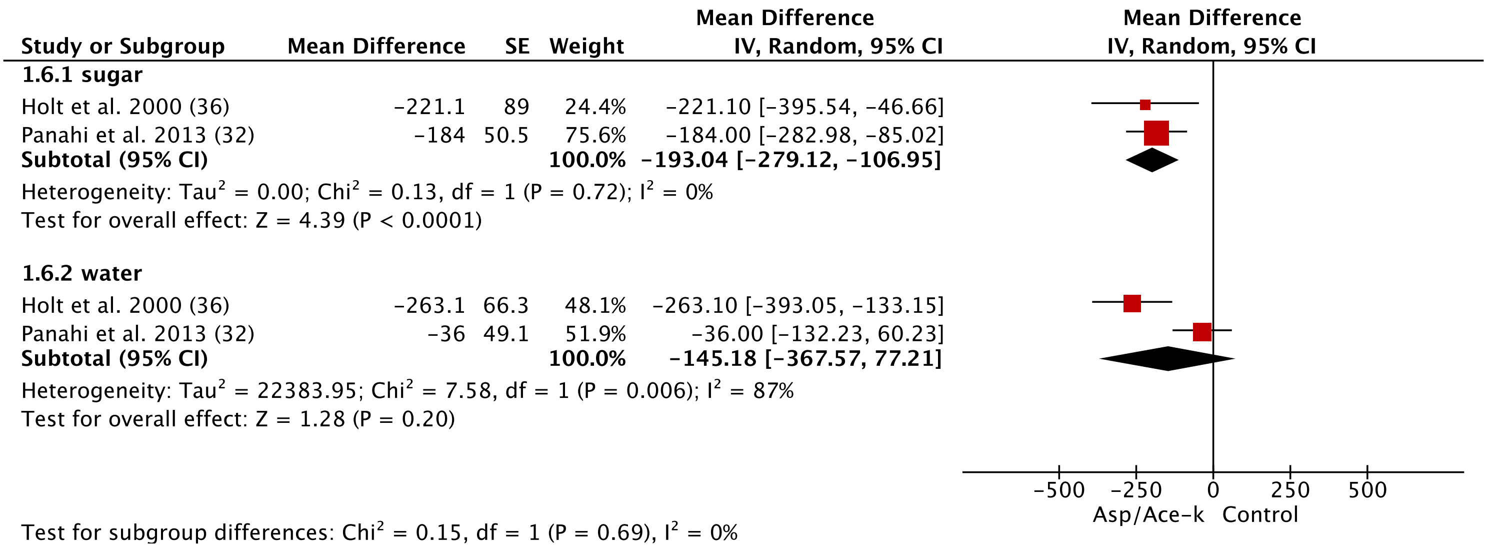

Test for subgroup differences: Chi² = 0.15, df = 1 (P = 0.69), I² = 0%

The combined effects of Aspartame and Acesulfame-k blends on appetite: a systematic review and meta-analysis of randomised clinical trials

Kirnjot Mehat

Supplemental Figure 2

Study or Subgroup	Mean Difference	SE	Weight	Mean Difference IV, Random, 95% CI
**1.7.1 v sugar**				
Olalde et al. 2013 (39)	−3.2	0.5441	30.7%	−3.20 [−4.27, −2.13]
Panahi et al. 2013 (32)	−1.25	0.314	34.1%	−1.25 [−1.87, −0.63]
Solomi et al. 2019 (38)	−0.04	0.2087	35.2%	−0.04 [−0.45, 0.37]
**Subtotal (95% CI)**			**100.0%**	**−1.42 [−2.96, 0.11]**
Heterogeneity: $Tau^2 = 1.70$; $Chi^2 = 34.15$, df = 2 ($P < 0.00001$); $I^2 = 94\%$				
Test for overall effect: Z = 1.82 (P = 0.07)				
**1.7.2 v water**				
Panahi et al. 2013 (32)	−0.25	0.241	46.2%	−0.25 [−0.72, 0.22]
Sylvetsky et al. 2016 (33)	0.1	0.221	53.8%	0.10 [−0.33, 0.53]
**Subtotal (95% CI)**			**100.0%**	**−0.06 [−0.40, 0.28]**
Heterogeneity: $Tau^2 = 0.01$; $Chi^2 = 1.15$, df = 1 (P = 0.28); $I^2 = 13\%$				
Test for overall effect: Z = 0.35 (P = 0.72)				

Mean Difference IV, Random, 95% CI

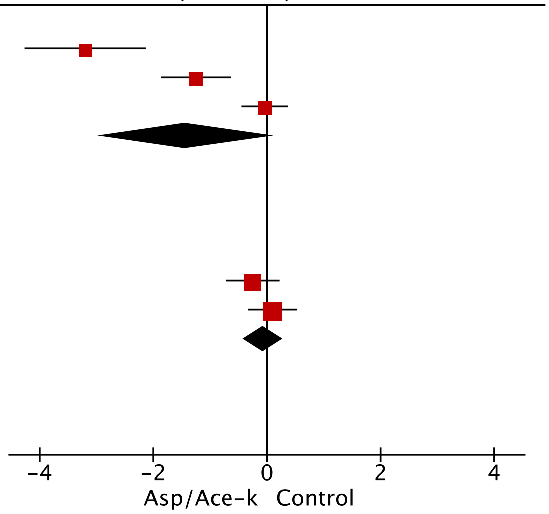

Test for subgroup differences: $Chi^2 = 2.88$, df = 1 (P = 0.09), $I^2 = 65.3\%$

The combined effects of Aspartame and Acesulfame-k blends on appetite: a systematic review and meta-analysis of randomised clinical trials

Kirnjot Mehat

Supplemental Figure 3

	Asp/Ace-K			Control				Mean Difference
**Study or Subgroup**	**Mean [mmol/L]**	**SD [mmol/L]**	**Total**	**Mean [mmol/L]**	**SD [mmol/L]**	**Total**	**Weight**	**IV, Random, 95% CI [mmol/L]**
**1.3.1 v sugar**								
Olalde et al. 2013 (39)	7.4	2	40	10.6	2.8	40	0.0%	−3.20 [−4.27, −2.13]
Panahi et al. 2013 (32)	7.35	1.6155	29	8.6	2.1541	29	48.9%	−1.25 [−2.23, −0.27]
Solomi et al. 2019 (38)	2.19	0.7589	10	2.23	1.2649	10	51.1%	−0.04 [−0.95, 0.87]
**Subtotal (95% CI)**			**39**			**39**	**100.0%**	**−0.63 [−1.82, 0.55]**
Heterogeneity: Tau^2 = 0.50; Chi^2 = 3.13, df = 1 (P = 0.08); I^2 = 68%								
Test for overall effect: Z = 1.04 (P = 0.30)								
**1.3.2 v water**								
Panahi et al. 2013 (32)	7.35	1.6155	29	7.6	1.3463	29	50.6%	−0.25 [−1.02, 0.52]
Sylvetsky et al. 2016 (33)	8.1	1.7	31	8	1.4	31	49.4%	0.10 [−0.68, 0.88]
**Subtotal (95% CI)**			**60**			**60**	**100.0%**	**−0.08 [−0.62, 0.47]**
Heterogeneity: Tau^2 = 0.00; Chi^2 = 0.40, df = 1 (P = 0.53); I^2 = 0%								
Test for overall effect: Z = 0.28 (P = 0.78)								

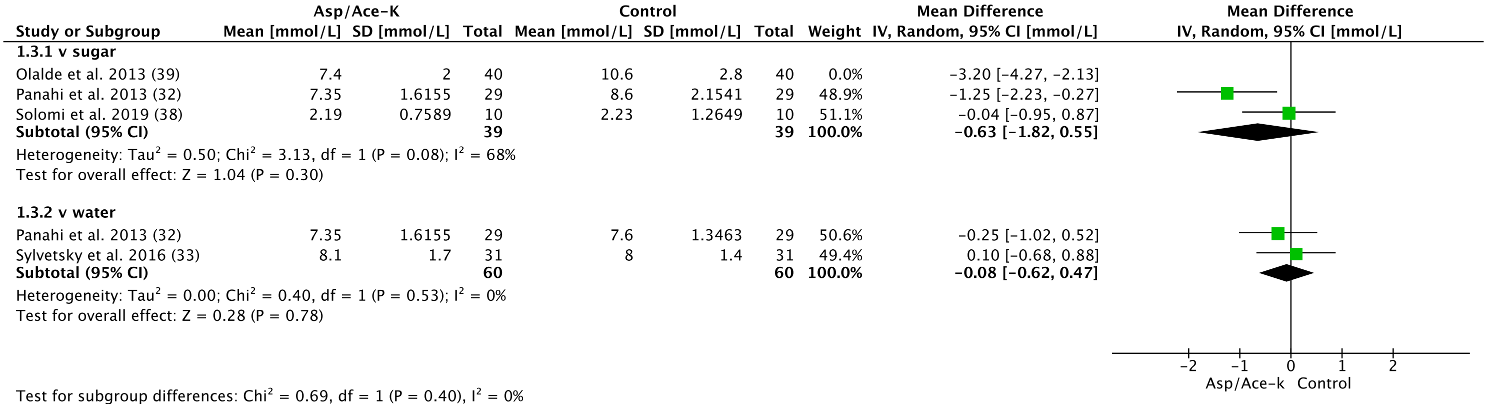

Test for subgroup differences: Chi^2 = 0.69, df = 1 (P = 0.40), I^2 = 0%
