# Supplementary material for: The Combined Effects of Aspartame and Acesulfame-K Blends on Appetite: A Systematic Review and Meta-Analysis of Randomized Clinical Trials
Source: Adv Nutr. 2022 Sep 3;13(6):2329–40. doi: 10.1093/advances/nmac072 (PMC9776645; doi:10.1093/advances/nmac072)
Supplement: nmac072_Supplemental_Files [file nmac072_supplemental_files.zip › Supplemental_Table_1.docx]

Supplemental Table 1

The combined effects of Aspartame and Acesulfame-k blend on appetite: a systematic review and meta-analysis of randomised clinical trials

Kirnjot Mehat

| Ovid MEDLINE(R) and Epub Ahead of Print, In-Process, In-Data-Review & Other Non-Indexed Citations and Daily <1946 to May 14, 2021> | | |
| --- | --- | --- |
| 1 | exp Non-Nutritive Sweeteners/ | 262 |
| 2 | artificial sweetener*.mp. | 1199 |
| 3 | sucralose.mp. | 804 |
| 4 | acesulfame potassium.mp. | 93 |
| 5 | neotame.mp. | 93 |
| 6 | cyclamate.mp. or Cyclamates/ | 885 |
| 7 | stevia.mp. or Stevia/ | 903 |
| 8 | aspartame.mp. or exp Aspartame/ | 1576 |
| 9 | saccharin.mp. or exp Saccharin/ | 5199 |
| 10 | 1 or 2 or 3 or 4 or 5 or 6 or 7 or 8 or 9 | 9035 |
| 11 | exp Glucagon-Like Peptide 1/ | 9108 |
| 12 | exp Cholecystokinin/ | 13545 |
| 13 | exp Gastrointestinal Hormones/ | 81007 |
| 14 | gastrointestinal hormone*.mp. | 8948 |
| 15 | exp Blood Glucose/ | 168569 |
| 16 | exp Insulin/ | 187646 |
| 17 | 11 or 12 or 13 or 14 or 15 or 16 | 365062 |
| 18 | appetite.mp. or exp Appetite/ | 35731 |
| 19 | exp Ghrelin/ or ghrelin.mp. | 11244 |
| 20 | glucose-dependent insulinotropic peptide.mp. or exp Gastric Inhibitory Polypeptide/ | 2863 |
| 21 | body mass index.mp. or exp Body Mass Index/ | 255598 |
| 22 | body weight.mp. or exp Body Weight/ | 617040 |
| 23 | energy intake.mp. or exp Energy Intake/ | 59078 |
| 24 | 17 or 18 or 19 or 20 or 21 or 22 or 23 | 1125481 |
| 25 | 10 and 24 | 1533 |
|  |  |  |
| Embase <1974 to 2021 Week 19> | | |
| 1 | exp Non-Nutritive Sweeteners/ | 292 |
| 2 | artificial sweetener*.mp. | 1514 |
| 3 | sucralose.mp. | 1604 |
| 4 | acesulfame potassium.mp. | 133 |
| 5 | neotame.mp. | 187 |
| 6 | cyclamate.mp. or Cyclamates/ | 1067 |
| 7 | stevia.mp. or Stevia/ | 1429 |
| 8 | aspartame.mp. or exp Aspartame/ | 2871 |
| 9 | saccharin.mp. or exp Saccharin/ | 7624 |
| 10 | 1 or 2 or 3 or 4 or 5 or 6 or 7 or 8 or 9 | 13380 |
| 11 | exp Glucagon-Like Peptide 1/ | 20019 |
| 12 | exp Cholecystokinin/ | 16171 |
| 13 | exp Gastrointestinal Hormones/ | 149968 |
| 14 | gastrointestinal hormone*.mp. | 6418 |
| 15 | exp Blood Glucose/ | 264598 |
| 16 | exp Insulin/ | 340446 |
| 17 | 11 or 12 or 13 or 14 or 15 or 16 | 640012 |
| 18 | exp somatostatin/ | 29897 |
| 19 | exp secretin/ | 8289 |
| 20 | exp glucagon like peptide 1/ | 20019 |
| 21 | exp food intake/ | 350732 |
| 22 | exp gastrin/ | 14144 |
| 23 | exp patient/ | 2597798 |
| 24 | exp obesity/ | 551171 |
| 25 | exp cholecystokinin/ | 16171 |
| 26 | exp gastrointestinal hormone/ | 149968 |
| 27 | gastrointestinal hormone*.mp. | 6418 |
| 28 | exp peptide YY/ | 4887 |
| 29 | 18 or 19 or 20 or 21 or 22 or 23 or 24 or 25 or 26 or 27 or 28 | 3493947 |
| 30 | appetite.mp. or exp Appetite/ | 71321 |
| 31 | glucose-dependent insulinotropic peptide.mp. or exp Gastric Inhibitory Polypeptide/ | 6112 |
| 32 | body mass index.mp. or exp Body Mass Index/ | 524291 |
| 33 | body weight.mp. or exp Body Weight/ | 664945 |
| 34 | energy intake.mp. or exp Energy Intake/ | 72577 |
| 35 | 17 or 29 or 30 or 31 or 32 or 33 or 34 | 4524590 |
| 36 | 10 and 35 | 3269 |
|  |  |  |
| Central COCHRANE | | |
| 1 | (artificial sweetener):ti,ab,kw (Word variations have been searched) with Publication Year from 1990 to 2021, in Trials | 248 |
| 2 | MeSH descriptor: [Sweetening Agents] explode all trees | 754 |
| 3 | MeSH descriptor: [Non-Nutritive Sweeteners] explode all trees | 35 |
| 4 | ("non-nutritive sweetener"):ti,ab,kw (Word variations have been searched) with Publication Year from 1990 to 2021, in Trials | 81 |
| 5 | (sucralose):ti,ab,kw (Word variations have been searched) with Publication Year from 1990 to 2021, in Trials | 215 |
| 6 | (acesulfame potassium):ti,ab,kw (Word variations have been searched) with Publication Year from 1990 to 2021, in Trials | 14 |
| 7 | (neotame):ti,ab,kw (Word variations have been searched) with Publication Year from 1990 to 2021, in Trials | 3 |
| 8 | (cyclamate):ti,ab,kw (Word variations have been searched) with Publication Year from 1990 to 2021, in Trials | 15 |
| 9 | MeSH descriptor: [Cyclamates] explode all trees | 6 |
| 10 | (stevia):ti,ab,kw (Word variations have been searched) with Publication Year from 1990 to 2021, in Trials | 83 |
| 11 | (aspartame):ti,ab,kw (Word variations have been searched) with Publication Year from 1990 to 2021, in Trials | 203 |
| 12 | MeSH descriptor: [Aspartame] explode all trees | 104 |
| 13 | (saccharine):ti,ab,kw (Word variations have been searched) with Publication Year from 1990 to 2021, in Trials | 259 |
| 14 | MeSH descriptor: [Saccharin] explode all trees | 55 |
| 15 | 1 or 2 or 3 or 4 or 5 or 6 or 7 or 8 or 9 or 10 or 11 or 12 or 13 or 14 | 1547 |
| 16 | (gastrointestinal hormone):ti,ab,kw (Word variations have been searched) with Publication Year from 1990 to 2021, in Trials | 1891 |
| 17 | MeSH descriptor: [Gastrointestinal Hormones] explode all trees | 3567 |
| 18 | (glucagon-like peptide 1):ti,ab,kw (Word variations have been searched) with Publication Year from 1990 to 2021, in Trials | 3752 |
| 19 | MeSH descriptor: [Glucagon-Like Peptide 1] explode all trees | 1791 |
| 20 | (Cholecystokinin):ti,ab,kw (Word variations have been searched) with Publication Year from 1990 to 2021, in Trials | 890 |
| 21 | MeSH descriptor: [Cholecystokinin] explode all trees | 426 |
| 22 | (Blood Glucose):ti,ab,kw (Word variations have been searched) with Publication Year from 1990 to 2021, in Trials | 48700 |
| 23 | MeSH descriptor: [Blood Glucose] explode all trees | 16312 |
| 24 | (insulin):ti,ab,kw (Word variations have been searched) with Publication Year from 1990 to 2021, in Trials | 60012 |
| 25 | MeSH descriptor: [Insulin] explode all trees | 13652 |
| 26 | (glucose-dependent insulinotropic peptide):ti,ab,kw (Word variations have been searched) with Publication Year from 1990 to 2021, in Trials | 476 |
| 27 | MeSH descriptor: [Gastric Inhibitory Polypeptide] explode all trees | 353 |
| 28 | (food intake):ti,ab,kw (Word variations have been searched) with Publication Year from 1990 to 2021, in Trials | 19164 |
| 29 | (appetite):ti,ab,kw (Word variations have been searched) with Publication Year from 1990 to 2021, in Trials | 9689 |
| 30 | MeSH descriptor: [Appetite] explode all trees | 1481 |
| 31 | (energy intake):ti,ab,kw (Word variations have been searched) with Publication Year from 1990 to 2021, in Trials | 12888 |
| 32 | MeSH descriptor: [Energy Intake] explode all trees | 5471 |
| 33 | ("body mass index"):ti,ab,kw (Word variations have been searched) with Publication Year from 1990 to 2021, in Trials | 39392 |
| 34 | MeSH descriptor: [Body Mass Index] explode all trees | 10316 |
| 35 | (body weight):ti,ab,kw (Word variations have been searched) with Publication Year from 1990 to 2021, in Trials | 63567 |
| 36 | MeSH descriptor: [Body Weight Changes] explode all trees | 8955 |
| 37 | 16 or 17 or 18 or 19 or 20 or 21 or 22 or 23 or 24 or 25 or 26 or 27 or 28 or 29 or 30 or 31 or 32 or 33 or 34 or 35 or 36 | 168164 |
| 38 | 15 and 37 | 783 |
|  |  |  |
| Web of Science Core Collection | | |
| 1 | (TS=(artificial sweetener*) )  AND LANGUAGE: (English) | 2003 |
| 2 | (TS=(Non-Nutritive Sweeteners) )  AND LANGUAGE: (English) | 308 |
| 3 | (TS=(sucralose) )  AND LANGUAGE: (English) | 1191 |
| 4 | (TS=(acesulfame potassium) )  AND LANGUAGE: (English) | 126 |
| 5 | (TS=(acesulfame-k) )  AND LANGUAGE: (English) | 416 |
| 6 | (TS=(neotame) )  AND LANGUAGE: (English) | 145 |
| 7 | (TS=(cyclamate*) )  AND LANGUAGE: (English) | 762 |
| 8 | (TS=(stevia) )  AND LANGUAGE: (English) | 1898 |
| 9 | (TS=(aspartame) )  AND LANGUAGE: (English) | 2414 |
| 10 | (TS=(saccharin) )  AND LANGUAGE: (English) | 5433 |
| 11 | 10 OR 9 OR 8 OR 7 OR 6 OR 5 OR 4 OR 3 OR 2 OR 1 | 11652 |
| 12 | (TS=(glucagon-like peptide 1) )  AND LANGUAGE: (English) | 18274 |
| 13 | (TS=(glp-1) )  AND LANGUAGE: (English) | 15223 |
| 14 | (TS=(cholecystokinin) )  AND LANGUAGE: (English) | 17773 |
| 15 | (TS=(gastrointestinal hormone*) )  AND LANGUAGE: (English) | 7866 |
| 16 | (TS=(blood glucose) )  AND LANGUAGE: (English) | 139797 |
| 17 | (TS=(insulin) )  AND LANGUAGE: (English) | 476913 |
| 18 | (TS=(food intake) )  AND LANGUAGE: (English) | 120116 |
| 19 | (TS=(energy intake) )  AND LANGUAGE: (English) | 69316 |
| 20 | (TS=(appetite) )  AND LANGUAGE: (English) | 26949 |
| 21 | (TS=(glucose-dependent insulinotropic peptide) )  AND LANGUAGE: (English) | 1874 |
| 22 | (TS=(gip) )  AND LANGUAGE: (English) | 4196 |
| 23 | (TS=(body weight) )  AND LANGUAGE: (English) | 336292 |
| 24 | (TS=(body mass index) )  AND LANGUAGE: (English) | 212950 |
| 25 | (TS=(bmi) )  AND LANGUAGE: (English) | 136779 |
| 26 | 25 OR 24 OR 23 OR 22 OR 21 OR 20 OR 19 OR 18 OR 17 OR 16 OR 15 OR 14 OR 13 OR 12 | 1117690 |
| 27 | 26 AND 11 | 1877 |

Supplemental Table 1. Complete Search Strategy for systematic review and meta-analysis.
